# Comparing hospitalized adult patients with chronic anorexia nervosa with versus without prior hospitalizations

**DOI:** 10.1186/s40337-024-01092-y

**Published:** 2024-09-04

**Authors:** Mary K. Martinelli, Colleen C. Schreyer, Angela S. Guarda

**Affiliations:** Department of Psychiatry and Behavioral Sciences, Johns Hopkins University School of Medicine, Baltimore, MD USA

**Keywords:** Anorexia nervosa, Inpatient treatment, Severe, Chronic, Eating disorders, Severe and enduring anorexia nervosa (SE-AN), Severe and enduring eating disorders (SEED), Hospitalization, Inpatient

## Abstract

**Background:**

Anorexia nervosa (AN) is a severe psychiatric disorder, from which recovery is often protracted. The role of prior specialized inpatient treatment on subsequent treatment attempts for adults with chronic AN and predictors of treatment response for severe and enduring AN (SE-AN) are needed to improve outcomes.

**Method:**

Participants (*N* = 135) with chronic AN (ill ≥7 years) admitted to an integrated inpatient-partial hospitalization eating disorders (ED) unit with prior ED hospitalization(s) (+ PH; *n* = 100) were compared to those without prior ED hospitalizations (-PH; *n* = 35) on admission characteristics (BMI, length of illness, outpatient ED treatment history, symptomatology (ED, anxiety, and depressive), history of suicide attempts or non-suicidal self-injury (NSSI)), treatment motivation and recovery self-efficacy, and discharge outcomes (discharge BMI, rate of weight gain, length of stay, clinical improvement).

**Results:**

Groups were similar with regard to age, years ill, and admission BMI. The + PH group had lower desired weight, lifetime nadir BMI and self-efficacy for normative eating, and higher state and trait anxiety than the -PH group. +PH were also more likely to endorse history of NSSI and suicide attempt. Regarding discharge outcomes, most patients achieved weight restoration at program discharge (mean discharge BMI = 19.8 kg/m^2^). Groups did not differ on rate of weight gain, likelihood of attending partial hospital, partial hospital length of stay, program discharge BMI, or likelihood of clinical improvement (*p*’s > 0.05) although inpatient length of stay was longer for the + PH group.

**Conclusions:**

Participants with chronic AN + PH exhibited more severe psychiatric comorbidity and lower self-efficacy for normative eating than AN -PH, however short-term discharge outcomes were similar. Future research should determine whether weight restoration and targeting comorbidities impacts relapse risk or need for rehospitalization among chronic and severe + PH. Despite similar illness durations, those with chronic AN -PH may be able to transition to partial hospital earlier. Conversely there is risk of undertreatment of chronic AN + PH given the recent shift promoting briefer self-directed admissions for adults with SE-AN. Research comparing + PH and -PH adults with chronic AN may facilitate efforts to individualize care and characterize relapse risk following intensive treatment.

## Abstract (plain language summary)

Some individuals with longstanding anorexia nervosa (AN) remain ill despite multiple attempts at intensive treatment. Others reach a high level of specialty care (e.g. inpatient or residential) for the first time only late in their illness. This study compared 100 hospitalized patients with chronic AN (ill ≥ 7 years) who previously received specialty inpatient eating disorder care to 35 hospitalized patients with chronic AN and no prior intensive treatment. Participants completed questionnaires at admission and weight change and hospital course were assessed at program discharge by chart review. At admission, individuals with prior hospitalizations reported greater difficulties with anxiety and suicidal behavior, lower confidence for changing their eating habits, and lower desired body weight compared to those with no prior inpatient treatment. Both groups had similar weight change and clinical improvement during treatment with mean discharge BMI consistent with weight restoration. These outcomes suggest equivalent short term improvement and weight restoration for individuals with chronic AN regardless of whether they previously received inpatient treatment and call into question whether the recent shift to brief admissions for those with chronic and severe AN may result for some in undertreatment, given that weight restoration remains the strongest predictor of long-term recovery.

## Background

Anorexia nervosa (AN) is a severe, complex psychiatric disorder from which recovery is often protracted. An estimated 20% of individuals with AN experience a chronic or persistent course [1], often referred to as severe and enduring anorexia nervosa (SE-AN) [2, 3]. Improving outcomes for SE-AN is critical given the medical sequelae, healthcare utilization costs, and high mortality associated with chronic AN [1, 4–6]. Research aimed at clarifying the etiology, maintenance, and course of those with chronic AN is urgently needed to improve treatment efficacy.

One challenge is the lack of an accepted definition of SE-AN, with current definitions emphasizing illness duration (7 years being the most common duration cutoff but some advocating for as few as 3 years) [2, 7]. Some definitions also require a history of non-response to evidence-based specialized eating disorder (ED) treatment [7, 8]. Problematically, however, most that include this criterion do not clearly specify how to define an adequate attempt at treatment [8]. Is a previous failure of outpatient cognitive-behavioral therapy (CBT) or a brief hospitalization for medical stabilization sufficient? Or should failure include at least one past admission and achievement of weight restoration in an intensive behavioral specialty treatment program for eating disorders? Or in the case of those who leave intensive treatment against medical advice or due to other family or financial concerns, how many premature discharges from intensive treatment constitute treatment failure? Few empirical studies of SE-AN have incorporated measures of treatment history when defining groups [9, 10], and results are mixed with some finding poorer outcomes among patients with previous ED treatment [11–13] and others not finding a relationship between history of previous treatment [14] or number of previous inpatient treatments [15] and outcomes.

The rationale for including treatment history in definitions of SE-AN is that individuals who do not improve, do not achieve remission, or quickly relapse following evidence-based treatment may represent a particularly vulnerable group for greater persistence of illness and for whom more targeted treatments are needed. Including prior treatment history in definitions of SE-AN is complicated, however. First, many individuals with diagnosable AN never seek treatment or receive inadequate treatment [16]. Others experience a long duration of untreated illness prior to engaging in evidence-based treatment [17] or engage in treatment, sometimes repeatedly, but drop out prematurely [18]. Potential obstacles to treatment include practical barriers (e.g., cost of treatment, wait times, geographic access to specialized care), stigma, low motivation to change, ambivalence or anxiety about intensive treatment, negative attitudes towards seeking help, low health literacy, and lack of social encouragement [19–23]. Additional barriers, such as low insight or lack of recognition of illness severity [19, 20, 23] may predict the severity or chronicity of AN [15, 24]. Thus, those who do not seek or complete treatment or who have a long duration of untreated illness may themselves represent a group more vulnerable to chronic, severe AN. In one retrospective study, individuals with a longer duration of untreated illness were less likely to have achieved remission at 20-year follow-up [25]. Finally, another challenge in incorporating prior treatment attempts in the definition of SE-AN is the issue of how best to classify type and intensity of treatment (e.g., brief medical stabilization admissions versus achieving full weight restoration in a multidisciplinary intensive behavioral specialty program for eating disorders).

Prior studies often compare individuals with a long duration of illness to those with early-stage AN (e.g., 26–29). Given that individuals with longer duration of illness are more likely to have engaged in prior treatment [11], it remains unclear whether long illness duration, history of treatment non-response, or both should be included in definitions of SE-AN. On one hand, a longer illness duration may make it more difficult to recover from AN regardless of prior treatment history due to factors such as greater genetic risk, greater physical complications [30], diminished social and psychological functioning [31, 32], and increased habit strength of AN behaviors [31, 33]. On the other hand, experiencing prior treatment non-response may uniquely influence subsequent treatment outcomes, by leading to diminished motivation or confidence in treatment [34, 35]. There may additionally be iatrogenic risks of treatment (e.g. traumatic experiences of care, or institutionalization) that contribute to persistent illness or treatment avoidance.

Investigating the role of prior treatment attempts independent of illness duration may help clarify definitional criteria and utility of the SE-AN label and inform treatment for severely and chronically ill adults with AN for whom treatment options are often more limited and evidence-based approaches sparse [9, 10]. This exploratory study of inpatients admitted to an integrated inpatient partial hospitalization ED program compared patients with long-term AN (ill ≥ 7 years) plus a prior history of inpatient ED behavioral treatment (+ PH) to patients with long-term AN seeking inpatient treatment for the first time (-PH). Groups were compared on admission characteristics, treatment outcomes, and hospital course. Given the exploratory nature of this study, no specific hypotheses were generated.

## Methods

### Participants and procedure

Data were collected as part of an ongoing, Institutional Review Board approved longitudinal study of response to intensive treatment in patients diagnosed with EDs. All first admissions to the Johns Hopkins Eating Disorder Inpatient-Partial Hospitalization Program between 2003 and 2022 were invited to participate. Eligible participants were individuals with AN who completed at least seven days of treatment, endorsed a length of illness ≥ 7 years at admission, provided informed consent, and completed questionnaires. Participants (*N* = 135) were divided into two groups: those who reported no previous specialized high level of ED care or hospitalization (inpatient or residential) at admission (-PH; *n* = 35) and those who endorsed a history of at least one prior specialized ED inpatient treatment (+ PH; *n* = 100).

Participants were diagnosed at hospital admission by trained raters, supervised by a licensed clinical psychologist, using the ED section of the Structured Clinical Interview for DSM-5 (SCID-5-RV) [36]. Participants admitted prior to 2015 were evaluated using the SCID-IV-TR, and diagnoses were later re-assessed using DSM-5 criteria. Participants completed a battery of self-report measures within the first week of admission. Clinical hospital course data were abstracted from the electronic medical record.

### Treatment protocol

The eating disorders program follows a structured behavioral treatment protocol delivered within a multidisciplinary integrated, inpatient-partial hospitalization stepdown program. Primary treatment targets include rapid weight restoration for underweight patients and normalization of eating behaviors. Cognitive behavioral therapy (CBT) and dialectical behavior therapy (DBT) informed psychotherapeutic interventions are delivered primarily in group format. Participants admitted below target weight were placed on a previously described standardized weight gain 100% meal-based nutritional protocol [37]. See Guarda et al. [38] for additional description of the treatment program.

### Measures

#### Demographic and clinical variables

Age, race, sex, marital status, income, education, and current employment were collected at admission via participant questionnaires. Measures pertaining to ED treatment history and illness course, including age of ED onset, length of illness (years), number of hospitalization(s) on a specialized ED unit prior to this admission, history of outpatient ED treatment, lifetime nadir body mass index (BMI), age at lifetime nadir BMI, and desired weight, were also gathered at admission. The + PH group was asked to report the length of stay (days) of their longest prior hospitalization for an ED.

#### Weight

Height and gowned morning weight at program admission and discharge were used to calculate admission and discharge BMI. Individual target weight was set as a four-pound range (1.8 kg) based on the patient’s age, sex, and height centered on a BMI of 20.5 kg/m^2^ for patients over age 25 [37]. For those aged 18–24, target weight was adjusted by subtracting one pound (0.45 kg) per year of age below 25.

#### Motivational factors

The University of Rhode Island Change Assessment (URICA) [39], a self-report measure of motivational readiness to change, was administered at admission. A 12-item version of the measure was adapted from the alcohol reduction version [40]. Responses were rated on a 5-point scale from 1 (Strongly Disagree) to 5 (Strongly Agree) and used to compute four Stage of Change subscales: pre-contemplation, contemplation, preparation or action, and maintenance. The URICA Readiness Score was computed by summing the contemplation, preparation or action, and maintenance subscales, and then subtracting the precontemplation subscale. Higher readiness scores indicate greater readiness to change.

#### Current ED symptomatology

The Eating Disorder Inventory-2 (EDI-2) [41] is a 91-item self-report questionnaire designed to measure psychological features and behavioral traits commonly associated with AN and bulimia nervosa (BN). The Drive for Thinness, Bulimia, and Body Dissatisfaction subscales were included in the current study. The EDI-2 has demonstrated good reliability and validity in individuals with EDs [42]. Internal consistencies in this study ranged from good to excellent (Drive for Thinness, α = 0.88; Bulimia, α = 0.92; Body Dissatisfaction, α = 0.91). The Eating Disorder Recovery Self-Efficacy Questionnaire (EDRSQ) [43] is a 23-item self-report measure of self-efficacy to cope with eating disorder behaviors and attitudes. The Normative Eating Self-Efficacy subscale measures confidence to eat without engaging in disordered behavior and without undue distress. The Body Image Self-Efficacy subscale measures confidence to maintain a realistic body image and not place undue influence of body weight and shape on self-esteem. The EDRSQ has demonstrated good validity and reliability [43, 44]. In the current study, internal consistencies for Normative Eating and Body Image were excellent (α = 0.96, α = 0.90). Frequency of Compensatory Behaviors was assessed via three self-report items at admission; participants rated their frequency of vomiting, laxative use to control weight, and excessive exercise over the past 8 weeks on a scale from 1 (Never) to 7 (More than once a day).

#### Comorbid psychopathology

The State-Trait Anxiety Inventory (STAI) [45] is a 40-item self-report scale measuring anxiety experienced in the moment (state anxiety) and as a stable personality trait (trait anxiety). The STAI has demonstrated good reliability and validity [46, 47]. The STAI state (STAI-S) and trait (STAI-T) subscale total raw scores demonstrated excellent internal consistencies in this study (α = 0.94, α = 0.91). Beck Depression Inventory-II (BDI-II) [48], a 21-item self-report measure of depression symptoms, has strong psychometric properties, including internal consistency and factor validity [49]. Internal consistency was excellent in the current study (Cronbach’s α = 0.91). Non-Suicidal Self-Injury (NSSI) was measured dichotomously as history of self-injurious behavior. Participants were asked if they had ever engaged in the following self-injurious behaviors (yes or no): cutting, burning, bruising, scratching. Due to low rates of endorsement for most of these behaviors and to avoid potential problems caused by zero-inflated data, the current study used only the responses for cutting to represent NSSI, as this was the most frequently endorsed behavior of those listed. Suicide Attempt was measured dichotomously with a yes or no to, “Have you ever attempted suicide in the past?”

#### Hospital course (discharge variables)

Length of Stay (days) for inpatient and partial hospital was calculated by subtracting admission date from discharge date for each participant. Reason for Discharge was dichotomized into “for clinical improvement” versus “not for clinical improvement” with the latter including discharge for non-compliance, elopement, financial reasons, patient/family reasons, or transfer. Partial Hospital Attendance was examined as percentage of participants per group transitioning to an integrated stepdown partial hospitalization program following inpatient treatment. Rate of Weight Gain was measured as kilograms gained per week and computed by dividing total weight gained in kilograms by the number of weeks spent on a weight gain nutritional protocol.

### Data analysis

Data were analyzed in SPSS version 28. Mann-Whitney *U* tests (continuous variables) and chi-square analyses (dichotomous variables) were conducted to explore differences between groups on demographic, clinical, and self-report measures at admission and discharge. Mann-Whitney *U* tests were chosen in lieu of independent-samples *t*-tests due to unequal sample sizes between groups and non-normality of variables. To test for differences between groups in discharge BMI, a linear regression model was conducted to control for admission BMI. Differences between groups were determined by examining the significance value and confidence intervals for the group variable coefficient. To account for non-normality and unequal sample sizes between groups, robust statistical methods for regression were employed (bootstrapping with confidence intervals and standard errors based on 1000 bootstrapped samples). All available data was utilized, and missing data is reported for each analysis.

## Results

### Sociodemographic characteristics

Descriptive data for participant sociodemographic characteristics are presented in Table 1. The majority of the sample was White (94.8%) and female (97.8%) with a mean age of 36.9 (*SD* = 11.2, range = 18–70) years. Participants with prior hospitalizations were more likely to be single or never married (*n* = 66, 67%) compared to those in the first-time hospitalization group (*n* = 14, 40%). Groups did not differ on age, sex, race, education, source of income, or current employment. The sample was highly educated with 90% of participants having at least some college education, however approximately half the sample reported their primary source of income as coming from social security or disability payments and only 28% earned income from salary/wages.

**Table 1:** Sociodemographic characteristics among participants with long-term AN with versus without prior hospitalizations (*N* = 135)

	-PH (*n* = 35)		+PH (*n* = 100)			
	*M*	*SD*	*M*	*SD*	Test statistic	*p*
Age (years)	38.06	12.05	36.54	10.99	*U* = 1582.00	0.399
	***n***	**%**	***n***	**%**		
Sex					*χ*^2^(1) = 2.65	0.103
Female	33	94.3	99	99.0		
Male	2	5.7	1	1.0		
Race^a^					*χ*^2^(1) = 3.75	0.053
African American	3	8.6	0	0.0		
White	31	88.6	97	97.0		
Asian	1	2.9	3	3.0		
Marital status					*χ*^2^(2) = 11.36	**0.003**
Single/never married	14	40.0	66	66.7		
Divorced or separated	7	20.0	19	19.2		
Married	14	40.0	14	14.1		
Education					*χ*^2^(3) = 3.60	0.308
≤ High school/GED	2	5.7	10	10.1		
Some college	13	37.1	38	38.4		
Bachelor’s degree	7	20.0	29	29.3		
≥ Master’s degree	13	37.1	22	22.2		
Income source					*χ*^2^(2) = 2.54	0.282
Salary/wages	11	31.4	26	26.3		
Parents/family/spouse	12	34.3	24	24.2		
SSI or SSDI	12	34.3	49	49.5		
Current employment					*χ*^2^(1) = 1.56	0.212
Unemployed	21	60.0	70	71.4		
Part- or Full-time	14	40.0	28	28.6		

*Note*. Significant *p*-values shown in bold. Percentages represent percent within group; *-PH*, no prior hospitalization; *+PH*, prior hospitalizations; *GED*, general education diploma; *SSI*, social security income; *SSDI*, social security disability insurance

^a^Asian and African American categories were collapsed for analysis due to insufficient cell size

### Weight variables and ED history at admission

Results of analyses comparing groups by weight and ED history at admission are presented in Table 2. Both groups had similar age and length of illness at admission. The mean admission BMI for the sample was 15.52 kg/m^2^ (*SD* = 1.98). Groups did not significantly differ on admission BMI, however participants in the + PH group endorsed a lower desired body weight (*Mdn* = 45.35 kg) compared to the -PH group (*Mdn* = 49.89 kg) and a lower lifetime nadir BMI (*Mdn* = 13.60) compared to the -PH group (*Mdn* = 15.01). Groups did not differ with regard to age of lifetime nadir BMI (*M* = 30.41, *SD* = 11.35), age of ED onset (*M* = 17.92, *SD* = 6.89), or length of illness (*M* = 19.01, *SD* = 9.84). The + PH group was significantly more likely to have received outpatient ED treatment, with 85% of the + PH group endorsing a history of outpatient ED treatment compared to 63% in the -PH group.

Participants in the + PH group were asked to respond to a multiple-choice question that queried them on the length of stay for their longest previous ED admission: 67% reported at least one prior admission lasting more than 30 days and only 8% reported that their longest prior stay was less than 15 days. These findings indicate that the majority of + PH participants had at least one prolonged hospitalization prior to the current admission.

### Motivation at admission

With regard to treatment motivation, no differences between groups were observed for URICA Readiness Score at admission (Table 2). The majority of the sample fell within the pre-contemplation or contemplation stages of change (*n* = 104, 77.6%) as opposed to the preparation or action stage of change (*n* = 30, 22.4%).

### ED symptomatology at admission

Results from analyses comparing groups by AN subtype and ED symptomatology at admission are displayed in Table 2. 64% of the sample was diagnosed with AN-BP (*n* = 86) and 36% was diagnosed with AN-R (*n* = 49). The proportion of AN-R subtype vs. AN-BP subtype did not differ by group. Groups did not differ on frequency of vomiting, laxative use, or excessive exercise over the past 8 weeks. The full sample reported vomiting (*M* = 3.45, *SD* = 2.63) and excessive exercise (*M* = 3.37, *SD* = 2.42) somewhere between several times per month and once per week, on average, and engaged in laxative use (*M* = 2.21, *SD* = 2.11) somewhere between once a month and several times per month, on average. Regarding self-report ED measures at admission, participants in the + PH group endorsed lower self-efficacy for normative eating (*Mdn* = 1.64) compared to participants in the -PH group (*Mdn* = 2.50). No differences between groups were observed for EDI-2 subscales or EDRSQ body image self-efficacy.

**Table 2:** Group comparison by clinical characteristics, ED history, and current ED symptomatology at admission

	-PH *n* = 35		+PH *n* = 100				
	*M*	*SD*	*M*	*SD*		Test statistic	*p*
Admission BMI	16.08	1.47	15.32	2.10		*U* = 1433.5	0.112
Length of illness, years	18.23	9.36	19.29	10.03		*U* = 1667.5	0.678
Age of ED onset	19.83	9.23	17.26	5.76		*U* = 1469.0	0.157
Desired weight (kg)^a^	48.48	7.11	45.20	6.21		*U* = 975.5	**0.024**
Lifetime nadir BMI^b^	14.70	1.64	13.31	2.14		*U* = 1009.0	**< 0.001**
Age at nadir BMI^c^	33.97	12.95	29.14	10.51		*U* = 1261.5	0.059
EDI-2^d^							
Drive for thinness	30.11	10.90	33.40	8.08		*U* = 1500.5	0.239
Bulimia	17.49	10.00	17.40	9.36		*U* = 1732.0	0.998
Body dissatisfaction	38.80	10.49	41.53	10.39		*U* = 1431.0	0.126
EDRSQ^d^							
Normative eating	2.50	1.17	1.93	0.95		*U* = 1263.5	.**017**
Body image	2.10	1.04	1.80	0.80		*U* = 1518.0	0.276
URICA Readiness^d^	10.72	1.61	10.40	2.05		*U* = 1599.0	0.498
Compensatory behavior							
Vomiting^b^	3.09	2.56	3.58	2.65		*U* = 1461.5	0.260
Laxative use^c^	2.11	2.00	2.24	2.16		*U* = 1611.5	0.828
Excessive exercise^e^	3.36	2.38	3.38	2.44		*U* = 1528.5	0.972
	***n***	***%***	***n***	***%***			
AN subtype						*χ*^2^(1) = 0.08	0.774
AN-R	12	34.3	37	37.0			
AN-BP	23	65.7	63	63.0			
Prior Outpatient Tx	22	62.9	85	85.0		*Χ*^2^(1) = 7.73	**0.005**

*Note*. Significant *p*-values shown in bold. *-PH*, no prior hospitalization; *+PH*, prior hospitalizations; *BMI*, body mass index (kg/m^2^); *ED*, eating disorder; *EDI*, Eating Disorder Inventory; *EDRSQ*, Eating Disorder Recovery Self-Efficacy Questionnaire; *URICA*, University of Rhode Island Change Assessment; *Tx*, Treatment

^a^*N* = 122, ^b^*N* = 130, ^c^*N* = 129, ^d^*N* = 134, ^e^*N* = 126

### Comorbid psychopathology at admission

Group comparisons on comorbid psychopathology are presented in Table 3. The majority of the sample (*n* = 75, 56%) met cutoffs for severe depression on the BDI-II (scores ≥ 29) at admission. No differences between groups were observed for BDI-II total (*M* = 29.88, *SD* = 12.55). With regard to anxiety, only 59% of the sample (*n* = 79) had data available on the STAI (60% in the -PH group and 58% in the + PH group) as the STAI was added to the study protocol in 2008. Using all available data, results show that the + PH group had significantly higher State Anxiety total scores (*Mdn* = 63) and Trait Anxiety total scores (*Mdn* = 65) compared to the -PH group (STAI-S: *Mdn* = 52; STAI-T: *Mdn* = 57). Of the participants with valid STAI data, approximately 47% and 65% fell into the clinically significant range (i.e., T-score ≥ 75) for state and trait anxiety, respectively. Participants in the + PH group were also significantly more likely to endorse a history of suicide attempt and NSSI (cutting) compared to the -PH group.

**Table 3:** Group comparison by comorbid psychopathology at admission

	-PH *n* = 35		+PH *n* = 100				
	*M*	*SD*	*M*	*SD*		Test statistic	*p*
BDI-II total^a^	26.86	12.58	30.95	12.43		*U* = 1413.0	0.105
STAI-State^b^	51.62	16.45	61.12	11.33		*U* = 406.0	**0.024**
STAI-Trait^b^	54.90	13.49	61.19	10.69		*U* = 431.0	**0.048**
	***n***	**%**	***n***	**%**			
NSSI - cutting^c^	3	9.4	31	35.2		*X*^2^(1) = 7.72	**0.005**
Suicide attempt^d^	4	12.1	38	43.2		*X*^2^(1) = 10.22	**0.001**

*Note*. Significant *p*-values shown in bold. *-PH*, no prior hospitalization; *+PH*, prior hospitalizations; *BDI*, Beck Depression Inventory; *STAI*, State-Trait Anxiety Inventory; *NSSI*, non-suicidal self-injury; *Hx*, History

^a^*N* = 134, ^b^*N* = 79, ^c^*N* = 120, ^d^*N* = 121

### Discharge outcomes and hospital course

Results from the regression model testing whether group membership at admission (+ PH or -PH) was associated with BMI at program discharge, controlling for admission BMI, are displayed in Table 4. Across the sample, the average BMI at discharge was 19.81 kg/m^2^ (*SD* = 1.99). Group membership at admission was not associated with BMI at discharge controlling for BMI at admission.

Results of Mann-Whitney *U* tests and Chi-square analyses comparing groups on discharge outcomes are presented in Table 5. No group differences were observed for rate of weight gain during treatment (inpatient: *M* = 1.91 kg/week, *SD* = 0.88; partial hospital: *M* = 1.29 kg/week, *SD* = 0.68), however, individuals in the + PH group had a longer length of inpatient stay (*Mdn* = 37 days) compared to those in the -PH group (*Mdn* = 30 days). No group differences were observed for partial hospital length of stay (*M* = 33.72 days, *SD* = 19.79) or likelihood of attending partial hospital, with 65% of the total sample transitioning from the inpatient to the partial hospitalization program. Groups also did not differ with regard to likelihood of discharge for clinical improvement, with 52% of the full sample classified as discharged for clinical improvement.

**Table 4:** Linear regression model testing group as a predictor of discharge BMI controlling admission BMI

	-PH *n* = 35		+PH *n* = 100				
	*M*	*SD*	*M*	*SD*	*b*	*SE b*	*p*
Discharge BMI	19.64	1.99	19.88	1.99			
Admission BMI					0.51 [0.34, 0.67]	0.09	< 0.001
Group					0.62 [-0.02, 1.30]	0.33	0.063

*Note*. Values in brackets indicate 95% confidence intervals based on 1000 bootstrap samples; *-PH*, no prior hospitalization; *+PH*, prior hospitalizations; *BMI*, body mass index (kg/m^2^)

**Table 5:** Group comparisons on discharge outcomes and hospital course

	-PH *n* = 35		+PH *n* = 100				
	*M*	*SD*	*M*	*SD*		Test statistic	*p*
Length of stay (days)							
Inpatient	32.03	16.15	43.25	26.38		*U* = 1328.00	**0.034**
Partial hospital^a^	36.00	18.53	32.95	20.28		*U* = 632.50	0.367
Rate of weight gain, kg/wk							
Inpatient^b^	1.83	0.90	1.94	0.88		*U* = 1621.00	0.572
Partial hospital^c^	2.95	1.27	2.79	1.58		*U* = 254.00	**0.916**
	***n***	**%**	***n***	**%**			
Attended partial hospital^a^	22	62.9	66	66.0		*X*^2^(1) = 0.11	0.737
Clinical improvement	20	57.1	50	50.0		*X*^2^(1) = 0.53	0.467

*Note*. Significant *p*-values in bold. *-PH*, no prior hospitalization; *+PH*, prior hospitalizations; *wk*, week

^a^*N* = 88, ^b^*N* = 134, ^c^*N* = 51

## Discussion

Effective treatments for adults with SE-AN are currently lacking and opinion is divided on how to best meet the needs of this chronically ill patient group characterized by high rates of morbidity, mortality, and functional impairment and lower quality of life [2, 4, 50]. Amongst psychiatric conditions, many patients with SE-AN account for disproportionate health care utilization costs [51, 52]. Long-term follow up studies, however, suggest that a majority of individuals with chronic AN will eventually recover, sometimes following several decades of illness, or multiple prolonged hospitalizations [53]. Weight restoration is the strongest predictor of recovery from AN, however relapse is not uncommon even among those discharged at a normative BMI [34]. It is unclear, however, whether successful weight restoration in a specialized inpatient behavioral treatment program confers longer term therapeutic advantages compared to brief admissions for medical stabilization or outpatient therapy alone for this group of patients. This is an important question for the field given the recent focus of the SE-AN literature on approaches to care that prioritize patient autonomy and choice, and focus on maximizing quality of life [3]. These priorities, along with economic forces and limited availability of intensive treatment, especially for severely and chronically ill adults with AN, have contributed to a shift favoring brief patient-directed admissions aimed at medical stabilization in lieu of repeated prolonged hospitalizations targeting full weight restoration and normalization of eating behavior. The purpose of the current study was to contribute to ongoing efforts within the field to clarify definitional criteria and utility of the SE-AN label to improve clinical decision-making and develop more effective treatments for chronic AN.

We compared voluntarily hospitalized adult patients with chronic AN who had previously received inpatient treatment to those hospitalized for the first time on admission characteristics, treatment outcomes, and hospital course. Sample descriptives and demographics were consistent with an adult SE-AN cohort. Average age was mid- to late thirties, average length of illness was 19 years, lifetime nadir BMI was reflective of extreme AN and the cohort had elevated rates of disability and functional impairment. Despite a high-level of educational attainment, the majority relied on social security or family or spousal financial support. Groups were similar at admission on age, duration of illness, BMI, ED and depressive symptomatology, and on motivation (readiness to change), although some cross-sectional group differences were observed suggestive of lower psychopathology in the -PH group on several admission variables including desired weight, lifetime nadir BMI, self-efficacy for normative eating, anxiety symptomatology, history of non-suicidal self-injury, and past suicide attempts. These differences may prove helpful in distinguishing illness course amongst those with chronic AN, independent of illness duration. For example, those with a lower lifetime nadir BMI in the + PH group may have been more likely to be identified or pressured into intensive treatment earlier by healthcare providers or social supports due to unstable labs or other medical or psychological symptoms. The -PH group were more likely to be married; having a spouse may reflect lower psychopathology or represent a protective factor by decreasing isolation and providing support. Conversely, a spouse may inadvertently accommodate the illness thereby facilitating treatment avoidance despite illness severity. The presence of greater psychiatric comorbidity (anxiety, NSSI, suicide) in the + PH vs. the –PH group is consistent with research indicating that comorbid mental health problems facilitate earlier help-seeking among individuals with EDs [16]. Higher psychiatric comorbidity may also explain the + PH group’s longer length of hospitalization despite similar admission BMI and rate of weight gain to the -PH group. Specific symptoms, for example greater behavioral dysregulation, comorbid anxiety, or self-injury, may have influenced the treatment team’s clinical assessment of readiness and safety to transition to a lower level of care. Despite these differences, both groups achieved similar short-term weight restoration and clinical outcomes at discharge.

Findings are consistent with previous research indicating that illness duration is not a good indicator for likelihood of weight restoration [26, 27] and suggest prior non-response to inpatient treatment in adults with SE-AN is not a strong predictor of subsequent short-term weight restoration and discharge outcomes. However, further research is clearly needed to clarify longer term outcomes (e.g., relapse rates 1-year post-discharge) following weight restoration among those with SE-AN.

Study findings have several important clinical and research implications. Some have called for alternative treatment approaches for individuals with SE-AN or for those with prior treatment non-response or relapsing illness [54]. These alternative approaches often emphasize harm reduction, focus on improving quality of life, and de-emphasize weight restoration and typically do not achieve weight restoration despite statistical increases in BMI [50, 55, 56]. More recently a focus on palliative care as a primary approach for some patients with SE-AN has also been promoted [57] and in rare cases recommendations have included consideration of hospice care, or in the extreme physician assisted suicide or medical aid in dying for some individuals with SE-AN [58]. The need for a primary palliative approach in SE-AN, however, remains controversial [59]. Principles of palliative care are already inherent in the competent practice of psychotherapy, including focus on quality of life and wellbeing as well as more targeted behavioral, supportive, and motivational approaches that promote clinical improvement and foster hope in eventual recovery.

Findings from this exploratory study suggest caution in deploying a harm reduction approach for individuals with chronic AN. Regardless of prior treatment history, most participants with long-term AN met criteria for weight restoration with an average discharge BMI of 19.8 kg/m^2^ across groups. The timing or likelihood of AN recovery can be difficult to predict, and the possibility of eventual recovery even in protracted cases of AN [11, 26, 27, 60] argues for maintaining an optimistic stance even for those who may not have responded to prior attempts at intensive treatment. As others have noted, we lack a reliable staging model for AN predictive of prognosis or of intensive treatment response [7, 61]. Similarly, a uniform definition for what comprises optimal evidence-based multidisciplinary inpatient treatment remains elusive and confounds any definition of SE-AN based on past treatment non-response. Both treatment length and context matter. Brief admissions for medical stabilization are unlikely to be as effective as achievement of full weight restoration in a multidisciplinary behavioral specialty treatment program for eating disorders. And for those who achieve weight restoration yet subsequently relapse, we do not know whether each successful cycle through treatment decreases both the gravitational pull of the eating disorder and the risk of relapse. AN is increasingly seen as a disorder of learning [31, 33] in which repeated behaviors become increasingly automatic and cue driven over time. Recovery, however, may also be a process of learning and repeated cycles of treatment associated with weight restoration may incrementally increase likelihood of eventual recovery. Qualitative interviews of recovered individuals with SE-AN and longitudinal studies are needed to assess this question. We do not know, for example, whether skills learned in prior treatment can be implemented by patients at a later date when motivation for recovery increases.

This study has several important limitations. Missing data for some variables as well as a limited sample size in the -PH group resulted in unequal sample sizes between groups. Measures were taken during data analyses to address these issues; however, results should be replicated with larger sample sizes and multisite research. Two central limitations relate to challenges presented by the lack of an accepted definition of SE-AN [2]. We based duration of illness ≥ 7 years on patients’ recall of the “age symptoms started to interfere with functioning”. Others, however, have defined illness onset as age at which all DSM diagnostic criteria are first met. Second, there is no accepted definition of what constitutes prior intensive treatment [3, 62]. We focused on history of inpatient ED treatment consistent with several extant studies [12, 63] but did not have information as to whether past treatments included achievement of weight restoration or were ended prematurely. This is especially significant given that the former remains the best predictor of recovery for AN [64]. Approximately two thirds of the -PH group had a history of outpatient ED treatment however close to one-third were treatment naïve. Future studies should also assess factors that may contribute to longer duration of untreated illness (e.g., lack of availability of specialized treatment units, inadequate insurance coverage) and/or individual factors (e.g., treatment anxiety or avoidance, low motivation).

## Conclusions

Although correlational and exploratory in nature, this study provides novel contributions to our understanding of intensive treatment for adults with chronic AN. Study findings help inform our understanding of differences in presenting characteristics and treatment course for individuals with chronic AN who have never been admitted compared to those with previous specialized ED hospitalizations. In the absence of a meaningful construct or definition of evidence-based intensive treatment, we believe these data support continued attempts to encourage patients to engage in active treatment with the goal of normalizing eating and weight control behaviors, treating co-occurring psychiatric conditions, restoring weight, and improving quality of life and functional level. Results support hopefulness for a good response to treatment even in those with chronic AN, whether or not they have received prior intensive treatment and reinforce the need for longitudinal studies of SE-AN that assess treatment course and predictors of outcome.

## Data Availability

The data that support the findings of this study are available on request from the corresponding author. The data are not publicly available due to privacy or ethical restrictions.

## Abbreviations

AN: Anorexia nervosa
AN-R: Anorexia nervosa, restricting type
AN-BP: Anorexia nervosa, binge/purge type
SE-AN: Severe and enduring anorexia nervosa
ED: Eating disorder
+PH: Prior hospitalization
-PH: No prior hospitalization
BMI: Body mass index
NSSI: Non-suicidal self-injury
